# Preferences of the peripheral olfactory system of Western Flower Thrips, *Frankliniella occidentalis* towards stereoisomers of common plant volatiles

**DOI:** 10.1007/s00049-014-0173-2

**Published:** 2014-10-18

**Authors:** Zayed S. Abdullah, Tariq M. Butt

**Affiliations:** Department of Biosciences, Swansea University, Singleton Park, Swansea, SA2 8PP UK

**Keywords:** Enantioselectivity, Electroantennogram, *Frankliniella occidentalis*, Olfaction, Polyphagous, Stereoisomers

## Abstract

Stereochemistry plays a significant role in structure–activity relationships of messenger chemicals. The ability to distinguish between enantiomers and geometric isomers, however, may be limited to certain stereoisomeric substances, depending on the receiver. In this study, we assessed the preference of the peripheral olfactometry system of Western Flower Thrips, *F. occidentalis* towards ubiquitously expressed host compounds, with a goal of establishing whether particular stereoisomers enhance host odour recognition. We demonstrate that the peripheral olfactory system of a highly polyphagous thysanopteran insect has evolved to become highly sensitive to a type of green leaf volatile, which is highly ubiquitous in the plant kingdom. We show that there is a significantly greater antennal response to the *cis* isomer, more so than the isomerisation by-product *trans*-3-hexen-1-ol. We demonstrate that the antennae of a highly polyphagous insect are capable of detecting common plant secondary metabolites in both enantiomeric forms.

**Electronic supplementary material:**

The online version of this article (doi:10.1007/s00049-014-0173-2) contains supplementary material, which is available to authorized users.

## Introduction

Insects utilize a peripheral olfactory system to sense chemical cues in the environment that are associated with mate and host recognition, as well as to avoid danger (Dethier 1947). Efficient localization of suitable hosts is paramount to species survival. Contrastingly, inability to identify unpalatable or toxic non-hosts severely decreases fitness. Sensilla situated on the insect antennae house olfactory receptor neurons (ORNs) activated by various chemical ligands from the external environment. The signals produced from these ORNs along with other sensory inputs travel to various processing regions of the brain, and a subsequent behavioural response then follows (Distler and Boeckh 1996; Galizia and Rössler 2010; Tolbert and Hildebrand 1981). Elucidating the nature of chemical ligand/receptor interactions at the antennal level is the first step in understanding the complex mechanisms of how insects process olfactory cues. Such understanding is important for the development of novel integrated pest management strategies that incorporate efficient use of semiochemicals, as well as discerning the vast use of olfactory cues in ecological systems.

Stereochemistry plays a significant role in structure–activity relationships of messenger chemicals (Mori 2002). The ability to distinguish between enantiomers and geometric isomers, however, may be limited to certain stereoisomeric substances, dependent on the receiver (Laska and Teubner 1999; Laska and Galizia 2001; Linster et al. 2001). In certain species of insects, chirality is important for the specificity of ORNs tuned to components of pheromones (Tumlinson et al. 1977; Leal 1996; Mori 1998). Various studies have determined enantiospecificity of the antennae responding to non-pheromonal odour compounds in insects of the orders Coleoptera, Lepidoptera and Hymenoptera (Wibe et al. 1998; Schorkopf et al. 2011; Stranden et al. 2002, 2003; Park et al. 2001). To our knowledge, however, no study has used antennal recordings to determine enantiomeric preferences in highly polyphagous thysanopteran insect pests towards common plant volatiles.

Western Flower Thrips, *Frankliniella occidentalis* P. (Thysanoptera: Thripidae), is a major agricultural and horticulture pest worldwide (Kirk 2002; Kirk and Terry 2003). The species has a broad host range of more than 500 species in 50 plant families (Yudin et al. 1986), making it a useful model for studies on polyphagous insect chemoreception. As a pest species, it causes damage and spoilage to a vast number of economically important plants through feeding, oviposition and spread of various plant diseases (Morse and Hoddle 2006). Given the species’ cryptic nature and small size, thrips can often remain undetected through quarantine control measures; hence, their spread has been facilitated by the increase in international plant movement (Kiritani 2001). As with most insect pests that have rapid generation periods and produce copious amounts of offspring, a major concern is the rapid development of resistance to conventional chemical pesticides (Bielza et al. 2007; Bielza 2008). This problem is accentuated by the fact that many pesticides have been withdrawn in the EU (EEC Directive 2009/128/EC), creating a need to develop alternative control methods.

In this study, we aim to assess the preference of the peripheral olfactometry system of *F. occidentalis* towards volatile compounds that are ubiquitously expressed in the plant kingdom. We further aim to establish whether particular enantiomeric or geometric isomers of electrophysiologically active compounds induce greater antennal outputs in comparison to their isomeric counterparts. Such knowledge will aid future research of chemoreception in highly polyphagous insects.

## Materials and methods

### Insects

A colony of *F. occidentalis*, kindly provided by Keele University, UK, was maintained on a bouquet of mixed cultivar chrysanthemum, *Dendranthema grandiflora*, potted in multi-purpose growing media (to allow for pupation of thrips), and kept on capillary matting inside ventilated Perspex cages (L 30 cm × W 30 cm × H 60 cm) at 25 ± 2 °C and L18: D6 photoperiod. The capillary matting was kept damp with distilled water. A fresh bouquet was placed in the rearing chamber every week, such that three bouquets were present in the rearing chamber at any one time. The oldest bouquet was removed upon addition of a new bouquet; this cycle provided optimal rearing conditions.

### Chemicals

**Fig. 1:**
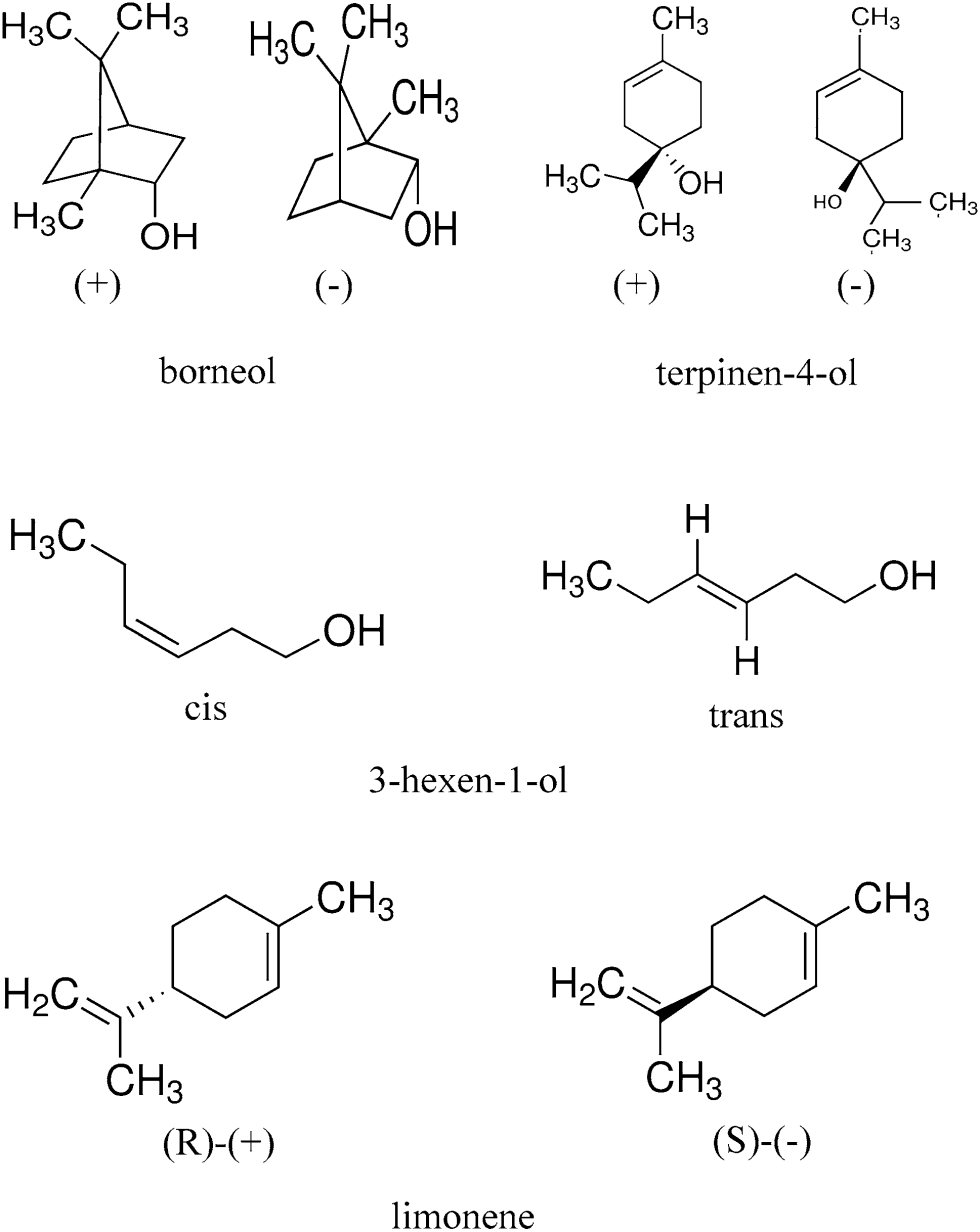
Chemical structures of some of the odour compounds used in this study. *Cis* and *trans*-3-hexen-1-ol are green leaf volatiles. Both (*R*) and (*S*)-limonene are monocyclic monoterpenes. (+) and (−)-terpinen-4-ol are also monocyclic monoterpenes. (+) and (−)-borneol are bicyclic monoterpenes

The purities and source of the chemicals used in this study are listed in Table 1. The stereoisomeric configurations of each compound are illustrated in Fig. 1.

**Table 1:** Source and purities of authentic chemical standards

Chemical	Source	Purity
Mineral oil (light oil)	Sigma Aldrich	Bioreagent, for molecular biology
1,8-cineole (eucalyptol)	Sigma Aldrich	≥99.0 % (GC)
*Cis*-3-hexen-1-ol	Sigma Aldrich	>98 %
*Trans*-3-hexen-1-ol	Sigma Aldrich	98 %
(*R*)-Limonene	Sigma Aldrich	97 %
(*S*)-Limonene	Sigma Aldrich	≥95 %
(+)-Terpinen-4-ol	Sigma Aldrich	Analytical standard
(−)-Terpinen-4-ol	Sigma Aldrich	≥95.0 %
(+)-Borneol	Sigma Aldrich	97 %
(−)-Borneol	Sigma Aldrich	Analytical standard

### Electrophysiological recordings

Electroantennograms (EAGs) of the antennae of *F. occidentalis* were adapted from a previous method that yielded good EAG recordings from thrips (Abdullah et al. 2014). The anterior portion of an adult female *F. occidentalis* was excised at the groove at which the mesonotum connects to the pronotum, and both antennae excised at the groove between antennal segments 7–8 (most distal to the head) to allow for better contact with the electrodes. The preparation was mounted by placing the proximal (head) end into the reference electrode and both antennae into the recording electrode using micromanipulators. The glass electrodes were filled with glucose-free Ringer’s solution (Maddrell 1969). Authentic standards were diluted in mineral oil (Sigma Aldrich; 1:100 v/v), and applied to strips of filter paper at a dose of 10 µl. A filter paper was placed in a disposable glass Pasteur pipet and allowed to sit for at least a minute to allow a constant release rate. The pipet was then attached to a 5 ml syringe via rubber tubing. The syringe plunger was depressed (over less than 2 s), expelling the volatile contents of the pipet into a purified airstream at a flow of 1 l/min through a glass tube (i.d. 120 mm) over the preparation. Separate syringes were used for each chemical. The EAG equipment consisted of a 10× gain universal probe (Syntech, Netherlands) and an IDAC 2 Signal Acquisition Processor (Syntech). 1,8-cineole standard was tested at the start of each replicate and these responses were used to normalize all test recordings to the largest response to the standard recorded in the experiment. To ensure that the amount of standard did not vary through the course of a block recording, a fresh standard preparation was tested at the end to ensure that EAG standard peaks were consistent in size. Responses to a mineral oil control were measured at the start and end of each preparation; these were normalized and then averaged for each block replicate. Fifteen recordings were made to each test compound using a minimum of five insects, recording three replicates per insect.

### Data and statistical analyses

Data were analysed with EAGPro Version 2 software, (Syntech). Data were square root transformed to obtain homogeneity of variance, and analysed using ANOVA. The model incorporated fixed effects of treatment, with both the day and the insect specimen used for each recording included as random factors. Means were compared using a Tukey post hoc test with SPSS software (IBM Corporation, USA).

## Results

### Response of antennae to individual plant compounds

**Fig. 2:**
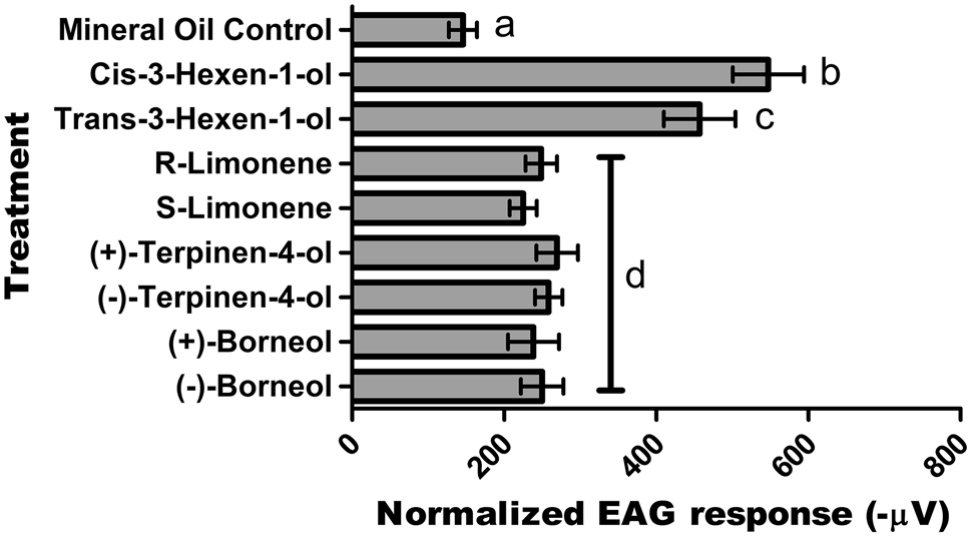
Mean electroantennogram (EAG) responses (normalized to 1,8-cineole) of adult female western flower thrips to stereoisomeric compounds (*N* = 15) (data presented as mean ± 95 % CI, with different letters representing samples that are significantly different; general linear model, Tukey post hoc test)

For representative EAG responses to each treatment, see Supplementary Fig. 1a–j. All chemicals evoked a significant electrophysiological response (*F*(8,121) = 91.796, *p* < 0.001) compared to the mineral oil control (Fig. 2). The highest depolarisations recorded were towards the green leaf volatiles (GLVs) *cis*-3-hexen-1-ol (*M* = 553 µV, SD = 13) and *trans*-3-hexen-1-ol (*M* = 463 µV, SD = 13). The depolarizations for the following compounds were: (*R*)-limonene (*M* = 255 µV, SD = 13), (*S*)-limonene (*M* = 231 µV, SD = 13), (+)-terpinen-4-ol (*M* = 270 µV, SD = 13), (−)-terpinen-4-ol (*M* = 259 µV, SD = 13), (+)-borneol (*M* = 239 µV, SD = 13) and (−)-borneol (*M* = 251 µV, SD = 13). These compounds all produced similar mean EAG depolarisations that were significantly larger than the depolarisations attributable to the mineral oil control treatment (*M* = 146 µV, SD = 13) (*F*(8,121) = 91.796, *p* < 0.001) but significantly lower than the GLVs *cis*-3-hexen-1-ol and *trans*-3-hexen-1-ol (*F*(8,121) = 91.796, *p* < 0.001).

### Capability of antennae to distinguish stereoisomers

Between the two geometric isomers, the compound *cis*-3-hexen-1-ol induced an antennal response that was significantly greater (*F*(8,121) = 91.796, *p* = 0.003) than *trans*-3-hexen-1-ol. Between enantiomers, there was no significant difference between (*R*)-limonene and (*S*)-limonene (*F*(8,121) = 91.796, *p* = 0.829), (+)-terpinen-4-ol and (−)-terpinen-4-ol (*F*(8,121) = 91.796, *p* = 0.999) or (+)-borneol and (−)-borneol (*F*(8,121) = 91.796, *p* = 0.996).

## Discussion

The largest mean depolarisation of *F. occidentalis* antennae was towards the GLVs *cis*-3-hexen-1-ol and *trans*-3-hexen-1-ol. GLVs are formed as a result of oxidative degradation of plant epicuticular waxes and are linked with host recognition via olfactory processes in phytophagous insects from a range of different orders (Visser 1983; Rajapakse et al. 2006; Bruce and Pickett 2011). *Cis*-3-hexen-1-ol induced a significantly larger mean EAG response in thrips compared to its geometric isomer *trans*-3-hexen-1-ol. *Cis*-3-hexen-1-ol is formed via the reduction of *cis*-3-hexenal, the isomerisation of which leads to the formation of *trans*-3-hexen-1-ol. The *trans* configuration is, therefore, less abundant in the emissions of either intact or wounded plants than the *cis* isomer (Matsui et al. 2012). Hence, from an evolutionary standpoint, a greater abundance of olfactory receptors better tuned to host volatiles of the more profuse geometric isomer would increase success of host seeking. This may explain the significantly larger mean EAG response to *cis*-3-hexen-1-ol.

Whilst there was a significant mean antennal EAG response of *F. occidentalis* towards all enantiomers of limonene, borneol or terpinen-4-ol compared to the mineral oil control, no significant difference was observed between enantiomers of each particular compound. There was a trend for (*R*)-limonene to produce a slight larger mean depolarisation compared to (*S*)-limonene, a trend that was also seen in previous work done on the parasitic wasp, *Microplitis croceipes* as well as the Coleopteran insects *Anthonomus grandis*, *Hylastinus obscures* and *Aegorhinus superciliosus* (Park et al. 2001; Palma et al. 2012; Dickens 1984; Mutis et al. 2010). The significant mean antennal EAG response of *F. occidentalis* towards (−)-borneol corroborates previous work; however, the same study found (−)-terpinen-4-ol to evoke a general EAG response that was not significantly different from the control (Abdullah et al. 2014). This may be explained by the fact that the aforementioned study used hexane as a control, which evoked more of an antennal response in *F. occidentalis* (presumably due to non-specific binding of receptors) than the mineral oil control treatment used in this study. All three enantiomeric compounds tested are ubiquitously expressed in the odour bouquet of a wide range of different plants (Williams and Whitten 1983; Borg-Karlson et al. 1987; Brunke et al. 1992; Dobson et al. 1997; Baser 2002; Flamini et al. 2002; Hadacek and Weber 2002), many of which *F. occidentalis* utilize as hosts (Yudin et al. 1986).

In conclusion, we have shown that the peripheral olfactory system of a highly polyphagous thysanopteran insect has evolved to become highly sensitive to a type of green leaf volatile, plant compounds that are highly ubiquitous in the plant kingdom. We have shown that there is a significantly greater antennal response to the *cis* isomer, more so than the isomerisation by-product *trans*-3-hexen-1-ol. Only further studies utilizing single cell recordings would determine the exact mechanisms that account for this response, such as whether the same ORNs are responding to both odours but with different sensitivity, or whether there are more ORNs of a certain type that binds the *cis*-3-hexen-1-ol. We demonstrate that the antennae of a highly polyphagous insect contain an array of ORNs capable of detecting common plant secondary metabolites in both enantiomeric forms.

As stereoisomeric compounds are common in many plant species, it would be interesting to know the stereoselectivity of *F. occidentalis* antennae towards even more plant compounds. Furthermore, it would be of great interest to test the antennal responses of highly polyphagous insects from different genera, families and orders against the common plant stereoisomers used in this study. This information would enhance our understanding of stereoselectivity at the peripheral olfactory level, thus aiding the development of more efficient lures or repellents that could be incorporated into integrated pest management systems against highly polyphagous insect pests.

## Electronic supplementary material

Below is the link to the electronic supplementary material.

Supplementary material 1 (DOCX 168 kb)
