# Supplementary material for: Preferences of the peripheral olfactory system of Western Flower Thrips, Frankliniella occidentalis towards stereoisomers of common plant volatiles
Source: Chemoecology. 2014 Oct 18;25(1):47–51. doi: 10.1007/s00049-014-0173-2 (PMC4289970; doi:10.1007/s00049-014-0173-2)
Supplement: Supplementary file 1 — Supplementary material 1 (DOCX 168 kb) [file 49_2014_173_MOESM1_ESM.docx]

**Supplementary 1**

**Figures S1a. Representative Electroantennogram responses for 1,8-cineole standard and mineral oil control.**


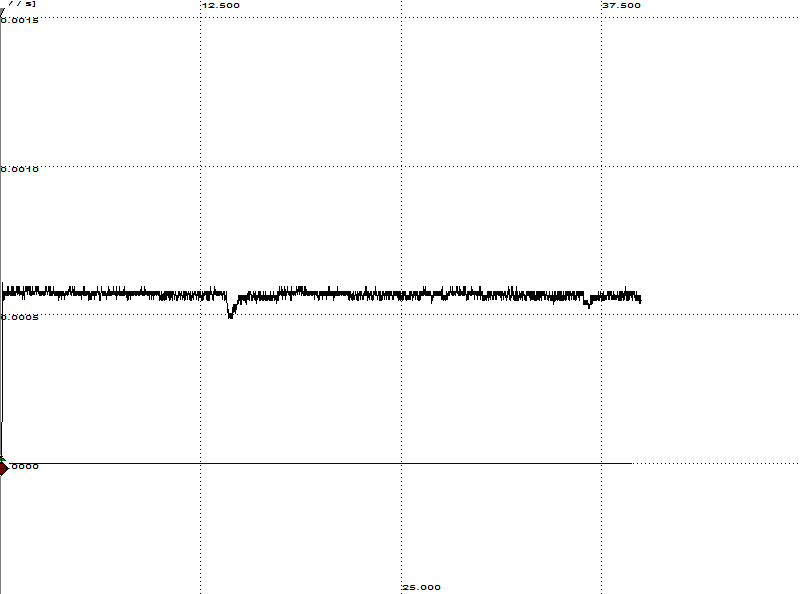


Mineral oil control

1,8-cineole

**Figures S1b. Representative Electroantennogram responses for 1,8-cineole standard and (*S*)-limonene.**


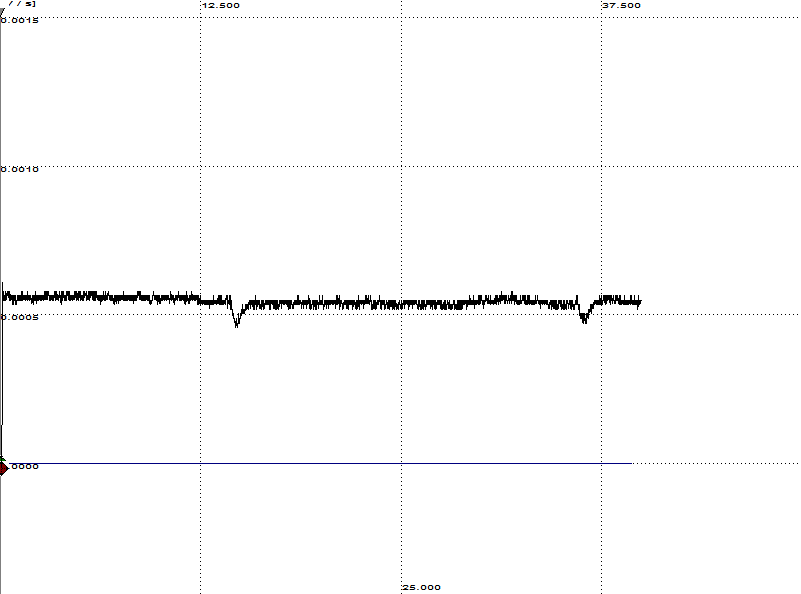


1,8-cineole

(*S*)-limonene

**Figures S1c. Representative Electroantennogram responses for 1,8-cineole standard and (*R*)-limonene.**


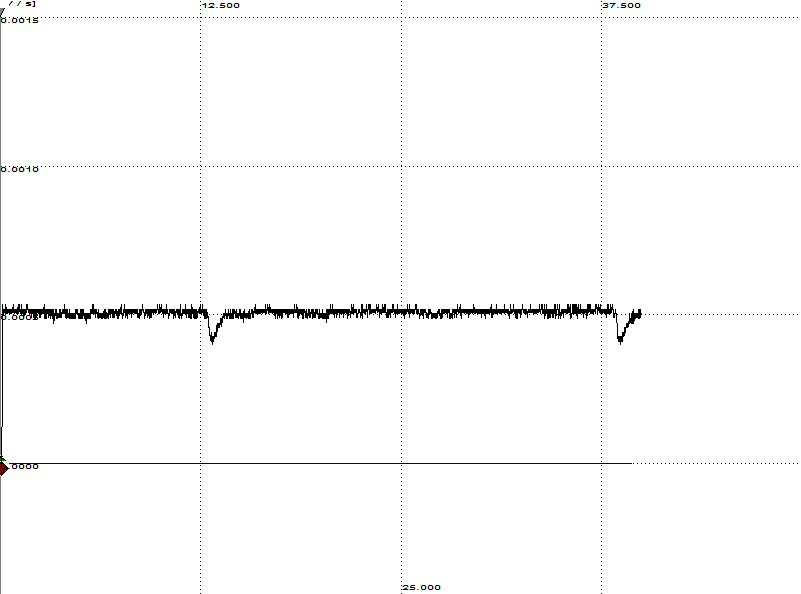


(*R*)-limonene

1,8-cineole

**Figures S1d. Representative Electroantennogram responses for 1,8-cineole standard and *cis*-3-hexen-1-ol.**


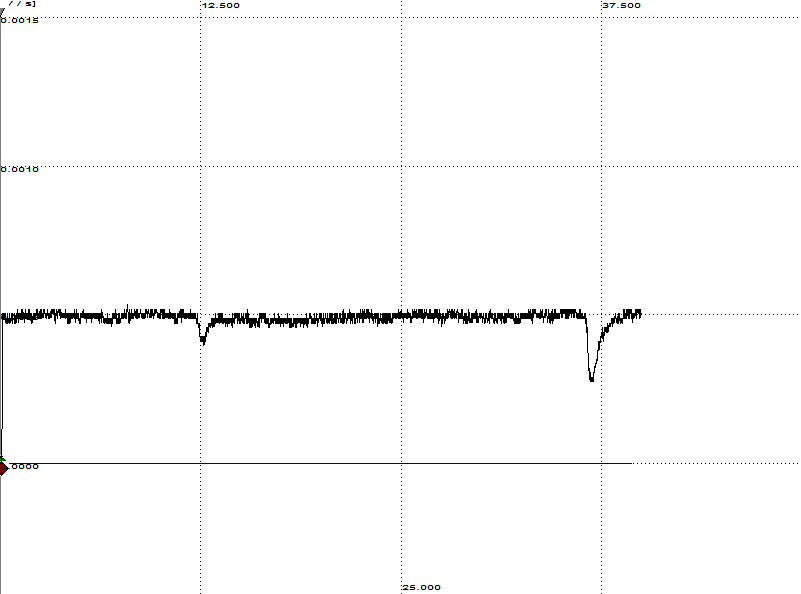


1,8-cineole

*cis*-3-hexen-1-ol

**Figures S1e. Representative Electroantennogram responses for 1,8-cineole standard and *trans*-3-hexen-1-ol.**


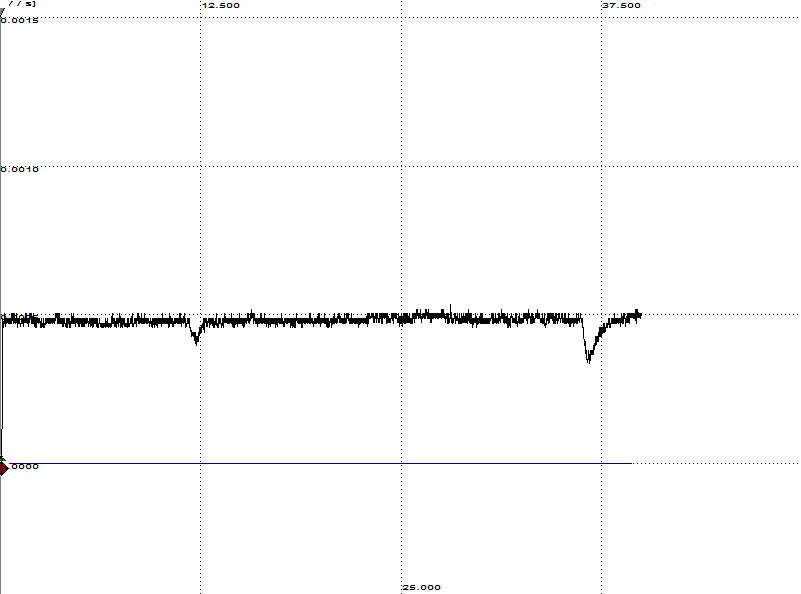


1,8-cineole

*trans-*3-hexen-1-ol

**Figures S1f. Representative Electroantennogram responses for 1,8-cineole standard and (+)-borneol.**


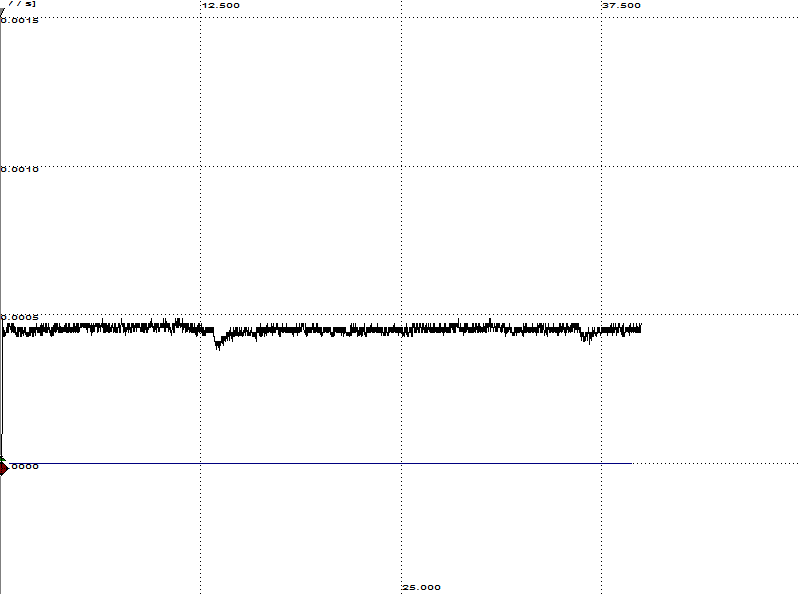


1,8-cineole

(+)-borneol

**Figures S1g. Representative Electroantennogram responses for 1,8-cineole standard and (-)-borneol.**


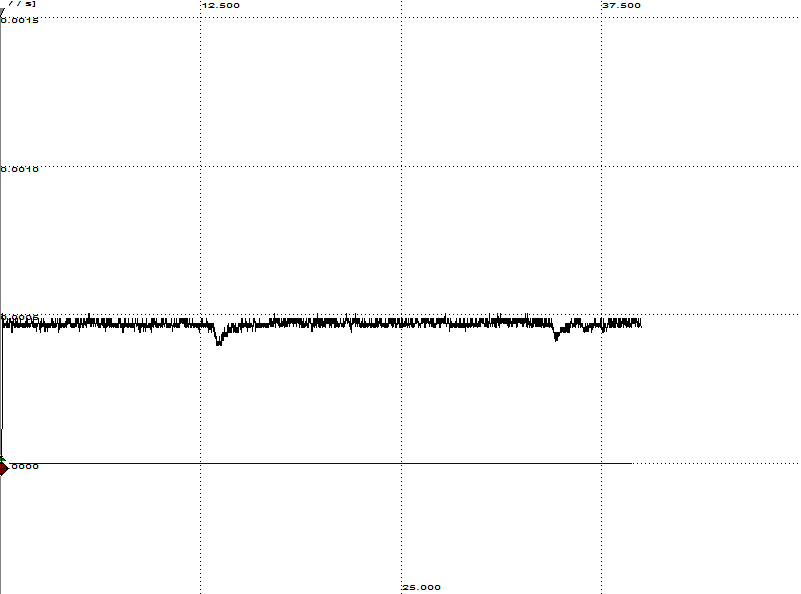


1,8-cineole

(-)-borneol

**Figures S1h. Representative Electroantennogram responses for 1,8-cineole standard and (+)-terpinen-4-ol.**


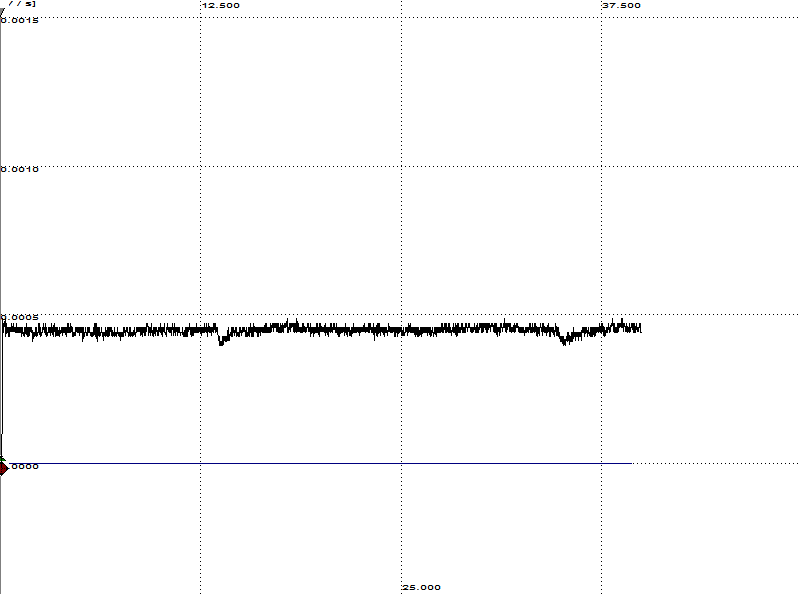


1,8-cineole

(+)-terpinen-4-ol

**Figures S1i. Representative Electroantennogram responses for 1,8-cineole standard and (-)-terpinen-4-ol.**

**
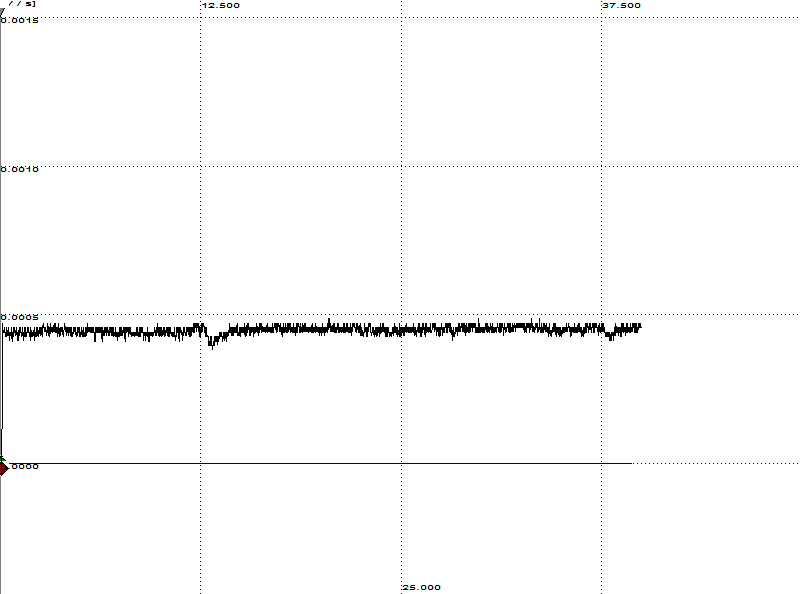
**

(-)-terpinen-4-ol

1,8-cineole

**Figures S1j. Standard at end of insect block replicate and fresh standard check.**


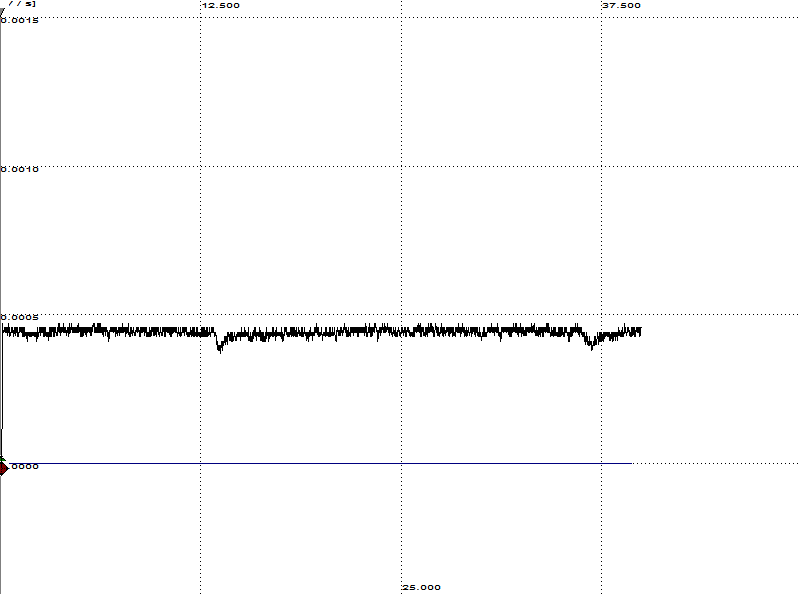


Reponse to freshly applied 1,8-cineole

Respone to 1,8-cineole at end of insect block replicate
